# Association Between Plasma Fibrinogen Level and the Risk of Myocardial Infarction With Non-Obstructive Coronary Arteries: A Retrospective Observational Study

**DOI:** 10.31083/RCM42845

**Published:** 2026-01-19

**Authors:** Di Li, Zongpeng Jing, Jijun Ding, Zongqian Xue

**Affiliations:** ^1^Department of Cardiology, Aoyang Hospital Affiliated to Jiangsu University, 215600 Zhangjiagang, Jiangsu, China

**Keywords:** fibrinogen, MINOCA, non-obstructive coronary artery disease, myocardial infarction

## Abstract

**Background:**

Myocardial infarction with non-obstructive coronary arteries (MINOCA) represents a heterogeneous clinical entity with an unclear pathophysiological basis. Fibrinogen is a key coagulation factor and inflammatory marker that has been associated with atherosclerotic burden in myocardial infarction (MI). However, the role of fibrinogen in MINOCA remains to be established. Therefore, this study aimed to investigate the association between plasma fibrinogen levels and the occurrence of MINOCA, and to evaluate the potential value of fibrinogen assessment in clinical characterization and early identification.

**Methods:**

This retrospective study initially screened 1759 patients diagnosed with acute myocardial infarction (AMI) who underwent coronary angiography. A total of 287 patients were analyzed after applying the inclusion and exclusion criteria: 87 with MINOCA and 200 with the MI alongside obstructive coronary artery disease (MI-CAD). A logistic regression analysis was used to assess the association between fibrinogen levels and MINOCA, with subgroup and interaction analyses performed. Receiver operating characteristic (ROC) and restricted cubic spline (RCS) analyses were conducted as supplementary evaluations.

**Results:**

Fibrinogen levels were significantly lower in the MINOCA group compared to the MI-CAD group (*p* = 0.005). Lower fibrinogen levels were independently associated with increased odds of MINOCA in the multivariate analysis (odds ratio (OR): 0.654, 95% confidence interval (CI): 0.483–0.885; *p* = 0.006). Quartile analysis revealed a significant inverse trend between fibrinogen levels and risk of MINOCA (*p* for trend = 0.006), which was further confirmed by a consistent dose–response relationship in the spline analysis (*p* for overall = 0.035; *p* for nonlinear = 0.590). The association remained robust across several subgroups. Fibrinogen alone showed a limited discriminative ability (area under the curve (AUC) = 0.605, 95% CI: 0.534–0.675; *p* = 0.005).

**Conclusions:**

Lower plasma fibrinogen levels were independently associated with the occurrence of MINOCA, suggesting a potential role in its pathophysiology and the early identification of this condition. Fibrinogen alone has limited discriminative utility; however, fibrinogen may contribute to multi-marker approaches for determining and managing MINOCA patients.

## 1. Introduction

Acute myocardial infarction (AMI) is primarily caused by atherosclerotic coronary artery disease (CAD), which leads to luminal narrowing or occlusion and subsequent myocardial ischemia or necrosis. Due to its abrupt onset and high fatality, AMI remains a significant public health concern worldwide. Early identification and timely intervention are essential for preserving myocardial tissue and improving patient outcomes.

In the 1980s, DeWood *et al*. [1] reported that approximately 90% of AMI patients undergoing coronary angiography had significant coronary obstruction, defined as ≥50% luminal stenosis. However, a small subset of patients, accounting for about 5%–15%, were found to have angiographically normal (0–30% stenosis) or only mildly stenotic (30%–50%) coronary arteries [2, 3, 4]. With the widespread application of invasive coronary angiography, such cases have become increasingly recognized. In response, the European Society of Cardiology (ESC) introduced the concept of myocardial infarction with non-obstructive coronary arteries (MINOCA) in 2017, along with diagnostic criteria [5]. Even with a standardized definition, the identification and management of MINOCA in clinical practice face many challenges due to its etiologic heterogeneity and complexity. In addition, although MINOCA patients do not exhibit significant coronary artery obstruction, their long-term prognosis is not benign. Emerging evidence indicates that MINOCA carries risks similar to CAD-related myocardial infarction (MI), including recurrent cardiovascular events and elevated in-hospital and long-term mortality [6]. These findings highlight the substantial cardiovascular risk associated with MINOCA and underscore the need for further investigation. However, most existing studies have focused on prognosis, with limited attention to the underlying pathophysiological mechanisms and related clinical factors. A few recent reports have investigated metabolic or inflammatory markers in non-obstructive coronary syndromes [7, 8], offering additional insights into this heterogeneous spectrum. Against this background, exploring readily available laboratory parameters may provide novel insights into the biological characteristics of MINOCA and support early recognition of this entity.

Fibrinogen is a key coagulation factor and acute-phase reactant that plays a critical role in thrombosis and inflammation. A previous study demonstrated a positive correlation between fibrinogen levels and the severity of CAD, as assessed by the Gensini score [9]. In addition, elevated fibrinogen has been closely associated with long-term mortality in patients with CAD or obstructive AMI [10, 11]. Nonetheless, the potential association between fibrinogen level and MINOCA has yet to be specifically investigated.

In this context, the present study aims to characterize the clinical features of MINOCA patients, evaluate the association between plasma fibrinogen level and the risk of MINOCA, and explore potential interactions with other clinical risk factors to improve clinical characterization and support early identification of MINOCA.

## 2. Methods

### 2.1 Study Population

A total of 1759 patients diagnosed with AMI [12] and undergoing coronary angiography at our regional chest pain center between October 2015 and September 2017 were consecutively enrolled. After excluding 256 patients who did not meet the inclusion criteria, 1503 patients were included in the study cohort.

**Fig. 1.**
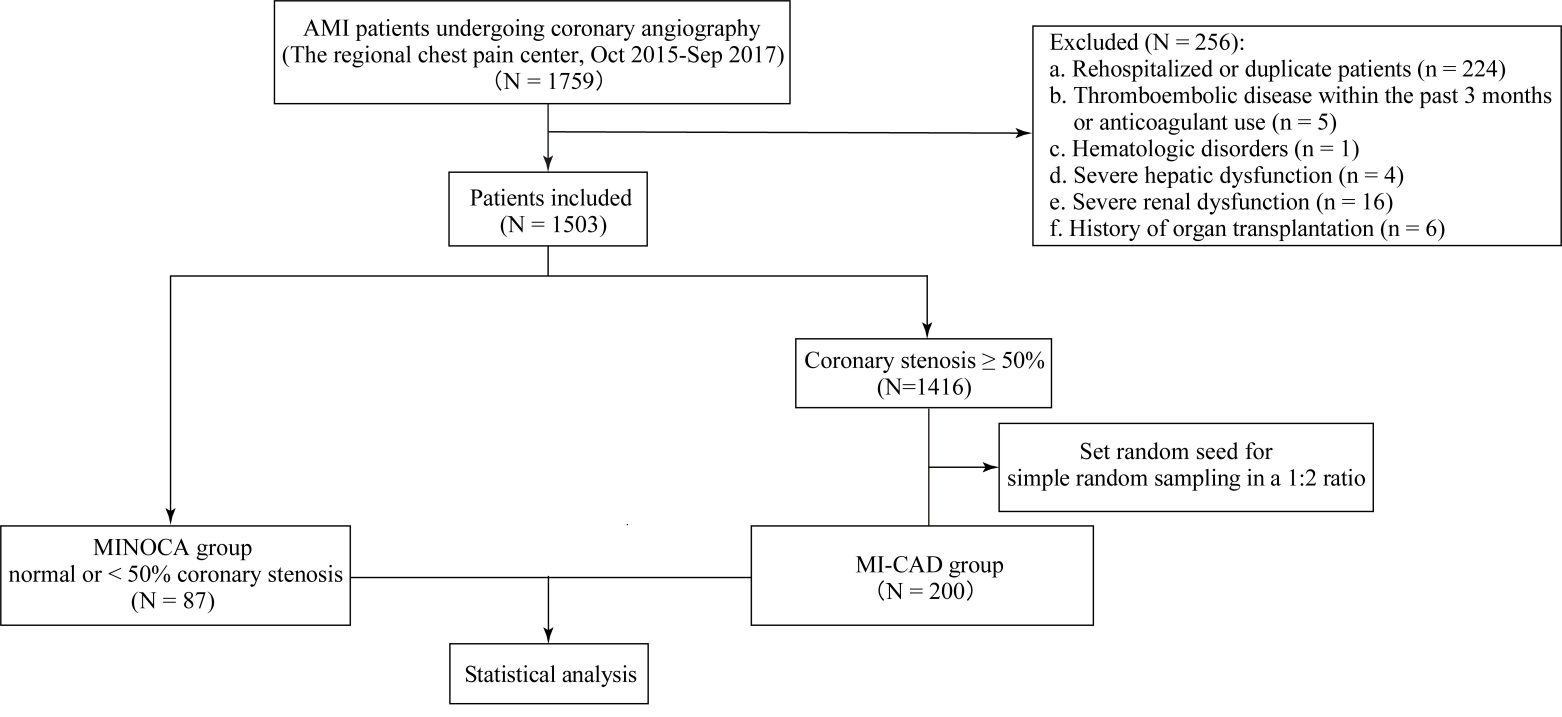
**Flowchart of patient selection and study design**. MINOCA, myocardial infarction with non-obstructive coronary arteries; MI-CAD, myocardial infarction with obstructive coronary artery disease; AMI, acute myocardial infarction.

After a comprehensive evaluation of clinical and cardiac magnetic resonance (CMR) imaging data to exclude overt non-ischemic myocardial injury (e.g., Takotsubo cardiomyopathy or myocarditis), a total of 87 patients with <50% coronary artery stenosis or normal coronary arteries were classified into the MINOCA group. Among the 1416 patients with ≥50% coronary artery stenosis, 200 were randomly selected at a 1:2 ratio to constitute the myocardial infarction with obstructive coronary artery disease (MI-CAD) group (see Fig. 1). Detailed definitions and inclusion/exclusion criteria are summarized in **Supplementary Table 1**.

### 2.2 Data Collection

The following data were collected from the hospital database: demographic information, medical history, blood test results on admission (including complete blood count and related hematological parameters, biochemical markers, coagulation profile, and troponin I), and left ventricular ejection fraction (LVEF) on admission as measured by transthoracic echocardiography (Philips Medical Systems iE33, Andover, MA, USA). The coagulation profile was based on test results obtained before the administration of antithrombotic drugs or coronary angiography on the day of admission. Data entry and verification were performed collaboratively by two investigators. Coronary angiography was performed using a Philips digital subtraction angiography (DSA) system (Philips Healthcare, Best, The Netherlands) after puncturing the right radial or femoral artery, as per the standards set by the American Heart Association (AHA) [13]. Two experienced interventional cardiologists reviewed and confirmed the results, with each independently performing over 50 coronary interventions annually. For patients with <50% coronary artery stenosis or normal coronary arteries, two experienced cardiologists evaluated the clinical records and CMR (MAGNETOM Skyra 3.0T, Siemens Healthcare, Erlangen, Germany) findings to exclude the possibility of non-ischemic myocardial injury.

### 2.3 Statistical Analysis

Normality was assessed using the Shapiro–Wilk test. Continuous variables with a normal distribution were expressed as the mean (standard deviation, SD), while non-normally distributed variables were expressed as the median (interquartile range, IQR). Categorical variables were presented as a frequency (percentage). Statistical tests included the *t*-test for continuous variables, the Chi-square or Fisher’s exact test for categorical variables, and the Mann–Whitney U test for non-normally distributed variables. All pairwise comparisons were conducted using one-way ANOVA or the Kruskal–Wallis test, followed by Bonferroni post hoc correction, or chi-square partitioning with a Bonferroni-adjusted significance threshold of *p* < 0.0083. To ensure consistency in sampling the MI-CAD group, an initial random number was generated using Stata version 16.0 (StataCorp LLC, College Station, TX, USA), followed by random sampling from the MI-CAD cohort to obtain a new sample for analysis. Logistic regression analysis was used to assess the relationship between MINOCA and various parameters. Variables with a *p*-value < 0.05 were included in the multivariate analysis. Receiver operating characteristic (ROC) analysis was conducted as a supplementary evaluation of discriminative ability, and restricted cubic spline (RCS) regression was used to examine potential dose–response relationships. Statistical significance was defined as a two-tailed *p*-value of <0.05. Data were analyzed using SPSS Statistics version 25.0 (IBM Corp., Armonk, NY, USA). R version 4.4.2 (R Foundation for Statistical Computing, Vienna, Austria), GraphPad Prism version 9 (GraphPad Software, San Diego, CA, USA), and Adobe Illustrator 2023 (Adobe Inc., San Jose, CA, USA) were used for data visualization.

## 3. Results

### 3.1 Baseline Characteristics

**Fig. 2.**
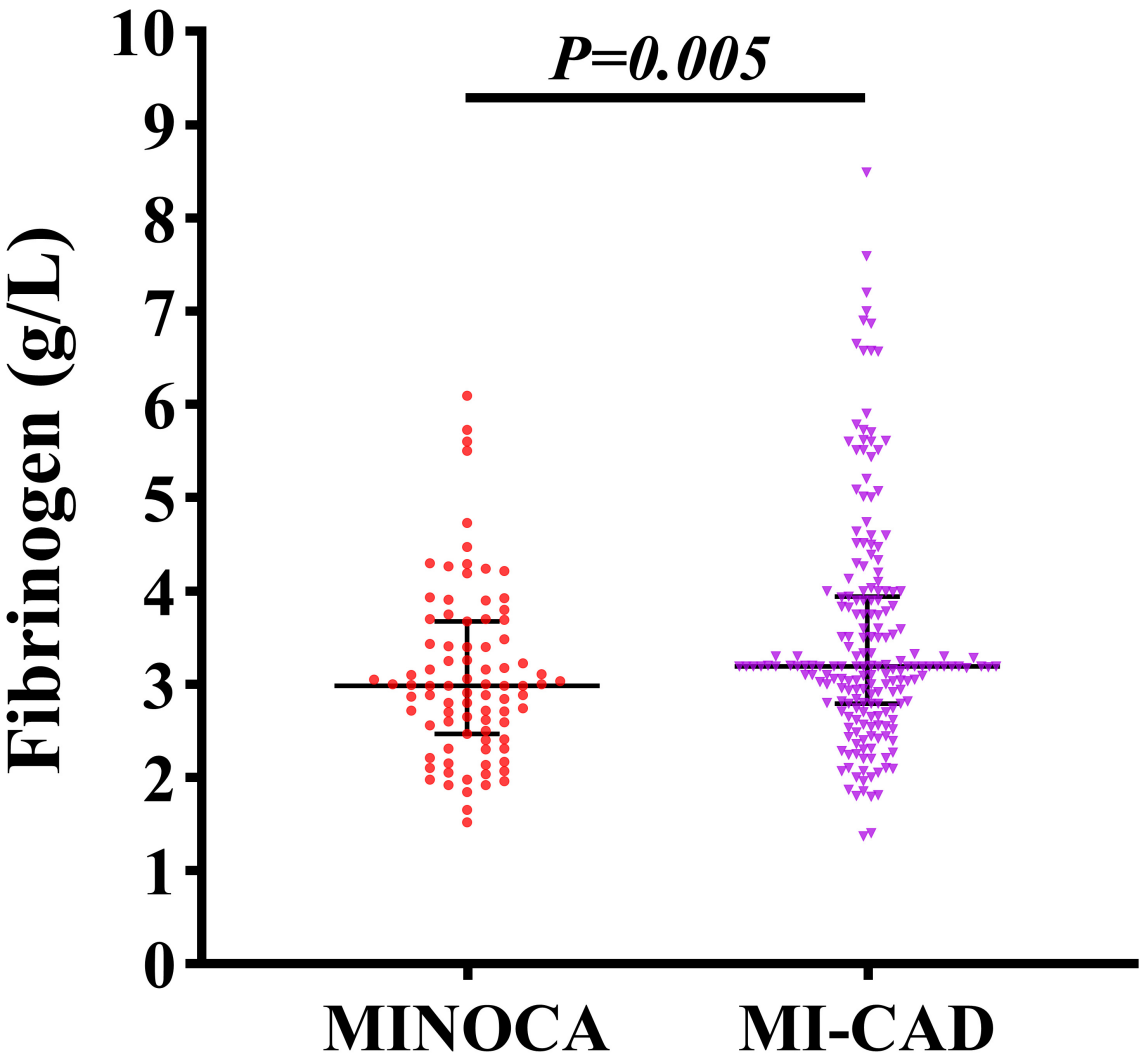
**Comparison of plasma fibrinogen levels between patients with MINOCA and MI-CAD**.

Of the 1759 AMI patients who underwent coronary angiography during the study period, 1503 were included in the cohort. Among them, 87 patients were identified as having MINOCA. For comparative analysis, 200 patients with MI-CAD were randomly selected at a 1:2 ratio. The baseline characteristics of the study population are presented in Table 1. No statistically significant differences were observed between the two groups in terms of age, sex, or body mass index (BMI). Similarly, heart rate, systolic blood pressure, and diastolic blood pressure were comparable across both groups. The prevalence of common cardiovascular comorbidities and risk factors, including smoking, alcohol consumption, hypertension, chronic obstructive pulmonary disease (COPD), family history of CAD, hyperthyroidism, and atrial fibrillation, did not differ significantly between the two groups. However, the prevalence of diabetes mellitus (*p* = 0.018) and ST-segment elevation (*p* = 0.006) on electrocardiogram was considerably lower in the MINOCA group compared to the MI-CAD group. Notably, plasma fibrinogen levels were significantly lower in patients with MINOCA compared to those with MI-CAD (*p* = 0.005), as shown in Fig. 2.

**Table 1.** **Baseline characteristics of the study subjects**.

	All subjects	MINOCA	MI-CAD	*p* value
	N = 287	N = 87	N = 200	
Age, years	64.0 (54.0, 73.0)	61.0 (52.0, 71.0)	66.0 (55.0, 73.0)	0.105
Male, n (%)	221 (77.0)	61 (70.1)	160 (80.0)	0.067
BMI, kg/m^2^	24.5 (21.2, 28.1)	24.1 (20.4, 27.1)	24.5 (21.5, 28.7)	0.307
Smoking, n (%)	164 (57.1)	45 (51.7)	119 (59.5)	0.221
Alcohol, n (%)	84 (29.3)	30 (34.5)	54 (27.0)	0.200
COPD, n (%)	23 (8.0)	7 (8.0)	16 (8.0)	0.989
Hypertension, n (%)	177 (61.7)	51 (58.6)	126 (63.0)	0.483
Diabetes mellitus, n (%)	65 (22.6)	12 (13.8)	53 (26.5)	0.018
Hyperthyroidism, n (%)	7 (2.4)	2 (2.3)	5 (2.5)	>0.999
Atrial fibrillation, n (%)	21 (7.3)	7 (8.0)	14 (7.0)	0.754
ST-segment elevation, n (%)	144 (50.2)	33 (37.9)	111 (55.5)	0.006
Family History of CAD, n (%)	22 (7.7)	7 (8.0)	15 (7.5)	0.873
Systolic BP, mmHg	145.0 (121.0, 162.5)	142.0 (121.5, 161.0)	148.0 (120.8, 163.0)	0.972
Diastolic BP, mmHg	78.0 (63.0, 91.0)	74.0 (59.0, 90.5)	79.0 (65.0, 91.0)	0.166
Heart rate (times/min)	90.0 (75.0, 111.0)	91.0 (75.0, 112.0)	90.0 (75.0, 109.0)	0.885

Data are presented as mean (SD), median (Q1, Q3), or number of patients (%), as appropriate. BMI, body mass index; COPD, chronic obstructive pulmonary disease; CAD, coronary artery disease; BP, blood pressure; SD, standard deviation; Q1, first quartile; Q3, third quartile.

### 3.2 Relationship Between Clinical Parameters and the Occurrence of MINOCA

As presented in Table 2, univariate logistic regression analysis identified four clinical variables that were significantly associated with the occurrence of MINOCA: white blood cell count (WBC), fibrinogen, cardiac troponin I, and LVEF. Specifically, the association with MINOCA were: WBC (OR: 0.836, 95% CI: 0.765–0.915, *p* < 0.001), fibrinogen (OR: 0.700, 95% CI: 0.540–0.908, *p* = 0.007), troponin I (OR: 0.946, 95% CI: 0.927–0.966, *p* < 0.001), LVEF (OR: 1.094, 95% CI: 1.061–1.128, *p* < 0.001). These variables were subsequently included in a multivariate logistic regression model to adjust for potential confounders. The results demonstrated that fibrinogen (OR: 0.654, 95% CI: 0.483–0.885, *p* = 0.006), troponin I (OR: 0.954, 95% CI: 0.934–0.974, *p* < 0.001), and LVEF (OR: 1.056, 95% CI: 1.022–1.091, *p* = 0.001) remained independently associated with the presence of MINOCA.

**Table 2.** **Logistic regression analysis of clinical parameters in MINOCA**.

Variables	Univariate analysis		Multivariate analysis	
	OR (95% CI)	*p* value	OR (95% CI)	*p* value
Age, years	0.587 (0.330–1.042)	0.069		
Sex	0.985 (0.967–1.004)	0.115		
BMI, kg/m^2^	0.969 (0.913–1.028)	0.298		
Smoking	0.729 (0.439–1.210)	0.222		
Alcohol	1.423 (0.828–2.445)	0.201		
WBC (×10^9^/L)	0.836 (0.765–0.915)	<0.001	0.998 (0.904–1.103)	0.971
PLT (×10^9^/L)	0.999 (0.996–1.002)	0.527		
MPV, fL	1.047 (0.871–1.257)	0.625		
PDW, %	1.000 (0.905–1.103)	0.992		
Plateletcrit, %	0.459 (0.019–11.334)	0.634		
Hemoglobin, g/L	0.993 (0.979–1.007)	0.301		
RDW, %	0.847 (0.608–1.179)	0.325		
Prothrombin Time, s	0.904 (0.725–1.128)	0.373		
Fibrinogen, g/L	0.700 (0.540–0.908)	0.007	0.654 (0.483–0.885)	0.006
Calcium, mmol/L	1.585 (0.398–6.313)	0.514		
Potassium, mmol/L	0.581 (0.308–1.098)	0.094		
BUN, mmol/L	0.957 (0.877–1.045)	0.331		
Uric acid, µmol/L	1.000 (0.998–1.002)	0.824		
Creatinine, µmol/L	0.992 (0.981–1.003)	0.140		
Albumin, g/L	0.978 (0.914–1.046)	0.507		
Globulin, g/L	0.990 (0.934–1.049)	0.732		
TC, mmol/L	0.953 (0.702–1.294)	0.757		
TG, mmol/L	0.894 (0.669–1.193)	0.447		
Troponin I, ng/mL	0.946 (0.927–0.966)	<0.001	0.954 (0.934–0.974)	<0.001
NT-proBNP, pg/mL	1.000 (1.000–1.000)	0.260		
LVEF, %	1.094 (1.061–1.128)	<0.001	1.056 (1.022–1.091)	0.001

WBC, white blood cell count; PLT, platelet count; MPV, mean platelet volume; PDW, platelet distribution width; RDW, red cell distribution width; BUN, blood urea nitrogen; TC, total cholesterol; TG, triglycerides; NT-proBNP, N-terminal pro-B-type natriuretic peptide; LVEF, left ventricular ejection fraction; OR, odds ratio; CI, confidence interval.

### 3.3 Characteristics of Study Participants According to Fibrinogen Quartiles

Participants were stratified into quartiles based on plasma fibrinogen levels: Q1 (≤2.66 g/L), Q2 (2.66–3.17 g/L), Q3 (3.17–3.84 g/L), and Q4 (≥3.84 g/L). The clinical characteristics across these quartiles are summarized in Table 3. A significant difference was observed in the prevalence of diabetes mellitus, which increased progressively across fibrinogen quartiles. Post hoc comparisons indicated substantial differences between Q2 and Q4. WBC counts also varied substantially between quartiles, with higher counts observed in Q3 and Q4 compared to Q2. Similarly, plateletcrit and prothrombin time showed statistically significant increases across fibrinogen quartiles, indicating enhanced prothrombotic profiles in higher fibrinogen groups. The levels of globulin and NT-proBNP were also positively associated with increasing fibrinogen quartiles. In contrast, LVEF was significantly lower in Q4 compared to Q2, indicating reduced cardiac function at elevated fibrinogen levels. Although the overall distribution of MINOCA across fibrinogen quartiles reached statistical significance, pairwise comparisons did not reveal significant differences. Nonetheless, a downward trend in the prevalence of MINOCA was observed with increasing fibrinogen levels, in agreement with the inverse association in multivariate analysis. No significant differences in other clinical variables were observed across the fibrinogen quartiles.

**Table 3.** **Characteristics of study participants according to fibrinogen quartiles**.

Variables	Fibrinogen				*p* value
	Q1 (≤2.66)	Q2 (2.66–3.17)	Q3 (3.17–3.84)	Q4 (≥3.84)	
	N = 72	N = 73	N = 71	N = 71	
Age, years	61.6 (13.4)	61.4 (13.9)	65.8 (11.6)	63.1 (14.3)	0.127
Male, n (%)	59 (81.9)	59 (80.8)	56 (78.9)	47 (66.2)	0.093
BMI, kg/m^2^	23.0 (19.8, 27.2)	24.7 (21.6, 27.8)	24.4 (21.2, 28.9)	25.8 (22.0, 28.7)	0.082
Smoking, n (%)	42 (58.3)	41 (56.2)	39 (54.9)	42 (59.2)	0.954
Alcohol, n (%)	21 (29.2)	22 (30.1)	18 (25.4)	23 (32.4)	0.828
Atrial fibrillation, n (%)	4 (5.6)	5 (6.8)	7 (9.9)	5 (7.0)	0.792
COPD, n (%)	5 (6.9)	5 (6.8)	7 (9.9)	6 (8.5)	0.898
Hypertension, n (%)	38 (52.8)	50 (68.5)	43 (60.6)	46 (64.8)	0.243
Diabetes mellitus, n (%)	15 (20.8) ^ac^	9 (12.3) ^a^	16 (22.5) ^ac^	25 (35.2) ^bc^	0.012
Hyperthyroidism, n (%)	0 (0)	1 (1.4)	3 (4.2)	3 (4.2)	0.238
ST-segment elevation, n (%)	39 (54.2)	30 (41.1)	41 (57.7)	34 (47.9)	0.200
Family History of CAD, n (%)	4 (5.6)	6 (8.2)	8 (11.3)	4 (5.6)	0.532
Systolic BP, mmHg	136.0 (119.0, 155.3)	148.0 (117.0, 167.0)	148.0 (117.0, 163.5)	148.0 (127.5, 163.0)	0.261
Diastolic BP, mmHg	75.0 (62.0, 95.0)	78.0 (65.0, 90.0)	80.0 (66.0, 93.0)	79.0 (60.5, 90.0)	0.634
Heart rate (times/min)	92.0 (75.0, 111.0)	90.0 (76.0, 112.0)	88.0 (76.0, 106.50)	91.0 (74.0, 112.5)	0.969
WBC (×10^9^/L)	7.9 (6.5, 10.6) ^ab^	7.5 (6.0, 9.8) ^b^	9.0 (7.2, 11.2) ^a^	9.5 (7.7, 12.1) ^a^	0.001
PLT (×10^9^/L)	196.5 (163.5, 222.5)	195.0 (155.0, 238.0)	196.0 (150.0, 238.5)	223.0 (164.0, 259.0)	0.102
MPV, fL	10.9 (10.3, 12.1)	10.8 (10.0, 11.7)	11.0 (10.2, 12.0)	10.9 (10.1, 11.9)	0.688
PDW, %	15.8 (12.7, 16.5)	15.9 (12.9, 16.5)	15.8 (13.4, 16.5)	15.7 (13.4, 16.3)	0.985
Plateletcrit, %	0.2 (0.1, 0.2) ^ac^	0.2 (0.1, 0.2) ^a^	0.2 (0.1, 0.2) ^ac^	0.2 (0.2, 0.3) ^bc^	0.044
Hemoglobin, g/L	136.4 ± 17.7	138.6 ± 21.7	134.6 ± 17.4	133.8 ± 15.2	0.397
RDW, %	12.8 (12.4, 13.3)	12.7 (12.4, 13.1)	12.9 (12.6, 13.4)	12.9 (12.5, 13.4)	0.257
Prothrombin Time, s	12.2 (11.6, 12.9) ^ab^	11.9 (11.3, 12.5) ^b^	12.2 (11.5, 12.6) ^b^	12.7 (12.0, 13.5) ^a^	<0.001
Calcium, mmol/L	2.2 (2.1, 2.3)	2.2 (2.2, 2.3)	2.2 (2.1, 2.3)	2.2 (2.1, 2.3)	0.195
Potassium, mmol/L	3.9 (3.8, 4.1)	3.7 (3.6, 4.1)	3.9 (3.7, 4.1)	3.9 (3.6, 4.1)	0.306
BUN, mmol/L	5.2 (4.5, 7.2)	5.3 (4.2, 6.9)	5.1 (4.2, 6.6)	5.6 (4.6, 7.7)	0.444
Uric acid, µmol/L	353.0 (295.8, 418.3)	369.0 (302.0, 412.0)	325.0 (260.5, 404.5)	375.0 (275.0, 471.5)	0.211
Creatinine, µmol/L	74.0 (64.0, 81.0)	73.5 (65.0, 86.0)	72.0 (62.0, 83.0)	75.0 (64.0, 92.5)	0.801
Albumin, g/L	39.7 (3.5)	40.2 (3.3)	39.3 (4.1)	38.7 (3.9)	0.119
Globulin, g/L	24.9 (22.4, 27.0) ^a^	25.9 (23.5, 28.4) ^ac^	25.6 (24.0, 28.3) ^ac^	28.1 (24.9, 31.3) ^bc^	<0.001
TC, mmol/L	4.5 (3.9, 5.1)	4.2 (3.5, 5.0)	4.5 (3.9, 5.1)	4.3 (3.8, 5.1)	0.338
TG, mmol/L	1.3 (1.0, 1.8)	1.4 (1.1, 2.4)	1.2 (0.9, 1.8)	1.4 (1.0, 1.8)	0.210
Troponin I, ng/mL	7.0 (1.4, 78.4)	4.6 (1.1, 32.1)	15.5 (1.6, 48.3)	10.0 (2.2, 31.1)	0.763
NT-proBNP, pg/mL	789.8 (234.5, 971.8) ^b^	971.8 (371.5, 1450.0) ^bc^	971.8 (553.0, 2100.0) ^ac^	1521.0 (971.8, 4302.0) ^a^	<0.001
LVEF, %	48.5 (45.0, 59.8) ^ac^	54.0 (48.0, 60.5) ^a^	51.0 (45.0, 60.0) ^ac^	48.0 (41.0, 57.0) ^bc^	0.024
MINOCA, n (%)	28 (38.9) ^a^	29 (39.7) ^a^	14 (19.7) ^a^	16 (22.5) ^a^	0.010

Data are presented as mean (SD), median (Q1, Q3), or number of patients (%), as appropriate. Q1, first quartile; Q2, second quartile; Q3, third quartile; Q4, fourth quartile. All pairwise comparisons were conducted using one-way ANOVA or the Kruskal-Wallis test, followed by Bonferroni post hoc correction, or chi-square partitioning with a Bonferroni-adjusted significance threshold of *p* < 0.0083. When an overall difference was significant among the four groups, superscript letters (a, b, c) indicate Bonferroni-corrected pairwise differences (*p* < 0.0083). Groups sharing the same letter do not differ significantly, while different letters denote significant differences.

### 3.4 Association Between Fibrinogen Quartiles and the Risk of MINOCA

The association between plasma fibrinogen levels and the risk of MINOCA was further evaluated by stratifying participants into quartiles and applying logistic regression models, as shown in Table 4. The lowest fibrinogen quartile (Q1, ≤2.66 g/L) served as the reference group. In the unadjusted model (Model 1), patients in Q3 (OR: 0.386, 95% CI: 0.182–0.819, *p* = 0.013) and Q4 (OR: 0.457, 95% CI: 0.220–0.950, *p* = 0.036) had significantly lower odds of MINOCA compared to Q1. This inverse trend remained statistically significant after adjusting for age and sex (Model 2), as well as for additional variables, including ST-segment elevation and diabetes mellitus (Model 3). The inverse association remained statistically significant in the fully adjusted model (Model 4), which accounted for age, sex, ST-segment elevation, diabetes mellitus, troponin I, and LVEF. The odds of MINOCA were significantly lower in Q3 (OR: 0.303, 95% CI: 0.123–0.745, *p* = 0.009) and Q4 (OR: 0.371, 95% CI: 0.153–0.902, *p* = 0.029) compared with Q1. A significant dose-response relationship was observed across quartiles in all models (*p* for trend < 0.01), indicating that higher fibrinogen levels were consistently associated with lower odds of MINOCA. To further evaluate the dose–response relationship, restricted cubic spline (RCS) analysis was performed, adjusted for age, sex, ST-segment elevation, diabetes mellitus, troponin I, and LVEF, which demonstrated a consistent linear association between fibrinogen level and the odds of MINOCA (*p* for overall = 0.035; *p* for nonlinear = 0.590) (**Supplementary Fig. 1**).

**Table 4.** **Association of fibrinogen quartiles with MINOCA**.

Models	OR (95% CI)				*p* for trend
	Q1 (≤2.66)	Q2 (2.66–3.17)	Q3 (3.17–3.84)	Q4 (≥3.84)	
	N = 72	N = 73	N = 71	N = 71	
Model 1	1.0	1.036 (0.532–2.017)	0.386 (0.182–0.819)	0.457 (0.220–0.950)	0.005
*p* value		0.918	0.013	0.036	
Model 2	1.0	1.023 (0.520–2.015)	0.400 (0.186–0.863)	0.396 (0.186–0.844)	0.003
*p* value		0.947	0.019	0.016	
Model 3	1.0	0.875 (0.435–1.760)	0.403 (0.184–0.881)	0.403 (0.185–0.878)	0.005
*p* value		0.708	0.023	0.022	
Model 4	1.0	0.722 (0.316–1.652)	0.303 (0.123–0.745)	0.371 (0.153–0.902)	0.006
*p* value		0.441	0.009	0.029	

Model 1: crude, no adjustment; Model 2: adjusting for age and sex; Model 3: adjusting for age, sex, ST-segment elevation, and diabetes mellitus; Model 4: adjusting for age, sex, ST-segment elevation, diabetes mellitus, troponin I, and left ventricular ejection fraction.

### 3.5 Stratified and Interaction Analyses of Fibrinogen and Covariates on the Risk of MINOCA

Stratified analyses were conducted across key clinical subgroups to explore the robustness of the association between fibrinogen and MINOCA. The results are summarized in Table 5. Statistically significant associations were observed among patients aged <65 years (OR: 0.557, 95% CI: 0.357–0.868), male patients (OR: 0.582, 95% CI: 0.388–0.873), those without ST-segment elevation (OR: 0.502, 95% CI: 0.313–0.806), non-diabetic patients (OR: 0.679, 95% CI: 0.482–0.957), patients with lower troponin I levels (OR: 0.547, 95% CI: 0.371–0.806), and those with preserved LVEF (OR: 0.532, 95% CI: 0.355–0.798). No statistically significant interactions were identified (*p* for interaction > 0.05 for all), indicating the association between lower fibrinogen levels and MINOCA was generally stable across subgroups.

**Table 5.** **Stratified analysis of associations between fibrinogen and MINOCA**.

Variables		OR (95% CI)	*p* for interaction
Age, years			0.385
	<65, N = 132	0.557 (0.357–0.868)	
	≥65, N =155	0.736 (0.471–1.151)	
Sex			0.477
	Male, N = 221	0.582 (0.388–0.873)	
	Female, N = 66	0.716 (0.416–1.231)	
ST-segment elevation			0.127
	Yes, N = 144	0.838 (0.533–1.316)	
	No, N = 143	0.502 (0.313–0.806)	
Diabetes mellitus			0.268
	Yes, N = 65	0.498 (0.198–1.247)	
	No, N = 222	0.679 (0.482–0.957)	
Troponin I, ng/mL			0.088
	<9.73, N = 144	0.547 (0.371–0.806)	
	≥9.73, N =143	1.208 (0.665–2.194)	
LVEF, %			0.108
	<50, N = 140	0.758 (0.421–1.365)	
	≥50, N =147	0.532 (0.355–0.798)	

Analyses were adjusted for age, sex, ST-segment elevation, diabetes mellitus, troponin I, and left ventricular ejection fraction, except when used as stratification variables. Interaction terms were also assessed for fibrinogen with these covariates, with adjustments for the same variables. Troponin I was categorized into two groups based on the overall median.

### 3.6 Predictive Performance of Fibrinogen for MINOCA

ROC analysis was performed as a supplementary evaluation, yielding an area under the curve (AUC) of 0.605 (95% CI: 0.534–0.675, *p* = 0.005), suggesting that fibrinogen alone has limited discriminatory ability for identifying MINOCA.

## 4. Discussion

### 4.1 Principal Findings

This study evaluated the association between plasma fibrinogen level and the risk of MINOCA. We found the fibrinogen level was significantly lower in patients with MINOCA compared to those with MI-CAD. In univariate and multivariate logistic regression analyses, lower fibrinogen level was independently associated with higher odds of MINOCA. Further analysis using fibrinogen quartiles revealed a consistent inverse trend, i.e., patients in higher fibrinogen quartiles exhibited a significantly lower risk of MINOCA, with a dose-response relationship confirmed across multiple adjusted models. This inverse association was further supported by RCS analysis, which demonstrated a significant overall linear trend. Subgroup analyses demonstrated this association remained stable across various clinical strata. Although fibrinogen alone had only limited predictive power, its significant independent association with MINOCA highlights its potential relevance as a pathophysiological or early identification marker.

### 4.2 Understanding the Clinical Complexity of MINOCA

MINOCA is now recognized as a distinct clinical entity within the AMI spectrum. Unlike obstructive CAD, which is characterized by significant luminal narrowing due to atherosclerotic plaque rupture, MINOCA occurs in the absence of obstructive epicardial lesions (defined as <50% stenosis). Nonetheless, it presents with typical features of MI, including ischemic symptoms, electrocardiographic changes, and elevated cardiac biomarkers [2, 3, 4]. Notably, growing evidence suggests it confers considerable risk for recurrent cardiovascular events and mortality, warranting greater clinical awareness and vigilance. The mechanisms underlying MINOCA are heterogeneous, encompassing both atherosclerotic and non-atherosclerotic processes. These range from plaque disruption that is undetectable by angiography to coronary microvascular dysfunction (CMD), epicardial coronary vasospasm, spontaneous coronary artery dissection (SCAD), and coronary embolism [2, 14, 15, 16, 17]. Non-invasive and angiographic assessments often fail to reveal the underlying substrate, necessitating the use of advanced intracoronary imaging techniques such as intravascular ultrasound (IVUS) or optical coherence tomography (OCT) for further evaluation [18, 19]. However, these techniques have limitations, including cost, invasiveness, operator dependence, and variable diagnostic yield depending on timing and lesion type [20]. Given the complex pathophysiology of MINOCA, its early recognition in real-world clinical practice remains suboptimal, which highlights the urgent need for accessible and reliable biomarkers to assist with timely detection and management in this heterogeneous population.

### 4.3 Biological Role and Clinical Relevance of Fibrinogen in Coronary Disease

Fibrinogen is a key plasma glycoprotein involved in both thrombosis and inflammation, two fundamental processes in the pathophysiology of CAD. Upon vascular injury, fibrinogen is cleaved by thrombin to form fibrin, the backbone of the thrombus, which facilitates platelet aggregation by binding to glycoprotein IIb/IIIa receptors on activated platelets [21]. Additionally, fibrinogen serves as an acute-phase reactant, and its level is elevated in systemic inflammatory states. High fibrinogen levels have been consistently associated with an increased risk of atherosclerotic cardiovascular disease and AMI. Several studies have demonstrated that higher fibrinogen concentrations correlate with prothrombotic clot characteristics, such as dense fibrin networks with reduced permeability and resistance to fibrinolysis, which are predictive of worse cardiovascular outcomes [22]. These findings suggest that fibrinogen is not merely a biomarker of inflammation but also an active contributor to thrombus formation and stability, thus playing a dual role in the development and progression of CAD. Given these mechanistic links, fibrinogen may serve as a valuable indicator of thrombotic risk and a potential target for cardiovascular risk stratification. Inevitably, as the mechanisms of MINOCA may involve vascular inflammation, microvascular dysfunction, and transient thrombosis with spontaneous lysis, it is reasonable to consider that fibrinogen could also play a role in this distinct form of CAD.

### 4.4 Potential Role of Fibrinogen in MINOCA and Pathophysiological Implications

Although fibrinogen has been extensively studied in obstructive MI, its relevance in the context of MINOCA is still not well defined. Unlike classic atherothrombotic mechanisms in MI-CAD, MINOCA encompasses diverse etiologies such as coronary vasospasm, microvascular dysfunction, and spontaneous thrombolysis, where the thrombotic burden may be less prominent or qualitatively different. A previous study suggested a higher prevalence of prothrombotic conditions, including antiphospholipid syndrome (APS), among MINOCA patients, particularly in younger and non-STEMI subgroups [23]. In contrast, another investigation found no significant differences in thrombin generation potential between MINOCA and MI-CAD patients, meaning the thrombotic burden in MINOCA is still an open question [24]. These uncertainties further obscure the role of fibrinogen in MINOCA. A recent meta-analysis reported elevated fibrinogen levels in MINOCA patients compared to those with MI-CAD [25]. On the contrary, the present study revealed that MINOCA patients had significantly lower fibrinogen concentrations compared to those with MI-CAD. This inverse association remained robust after multivariable adjustment and across multiple subgroups. One reason for this discrepancy may be the diagnostic approach. Prior study used a working diagnosis of MINOCA, which could include cases of non-ischemic myocardial injury, such as myocarditis or Takotsubo syndrome. In our study, MINOCA was further confirmed by CMR, thereby minimizing confounding factors and providing a clearer picture of fibrinogen levels in this group. Several plausible mechanisms could underlie our observation. First, in the absence of overt plaque rupture or large-vessel thrombosis, as seen in many MINOCA cases, there may be less acute-phase stimulation or fibrinogen consumption. Second, the inflammatory response may be less intense in vasospasm- or microvascular-driven MINOCA than in MI-CAD. Third, subclinical coagulopathies, endothelial dysfunction, or early thrombus lysis may contribute to a lower circulating fibrinogen profile. Collectively, these mechanisms suggest that low fibrinogen levels may reflect a distinct biological phenotype within the MINOCA population, potentially related to non-obstructive and non-thrombotic pathways.

### 4.5 Sex Differences in MINOCA: Current Evidence and Study Insights

Previous studies have consistently reported that MINOCA disproportionately affects females [2, 3, 4]. Several underlying mechanisms have been proposed to contribute to this sex-specific vulnerability, including CMD, endothelial dysfunction, and hormonal influences, such as estrogen-mediated vasomotor modulation. Female patients with MINOCA are also more likely to exhibit non-classical presentations, such as atypical chest pain or emotional triggers, and may have a higher prevalence of coronary vasospasm than male patients. In our study, although the proportion of female patients in the MINOCA group was higher than in the MI-CAD group (29.9% vs. 20.0%), the difference did not reach statistical significance (*p* = 0.067). Our findings appear to contradict the prevailing notion of a female predominance in MINOCA. Several factors may underlie this finding in our cohort. First, the relatively modest sample size, particularly for the female subgroup (n = 66), may have limited the statistical power to detect sex-based differences. Second, the use of CMR-confirmed criteria rather than a working diagnosis, while enhancing diagnostic specificity, may have diluted the apparent female predominance. Third, our cohort may underrepresent specific female-predominant pathophysiological subtypes of MINOCA, such as SCAD or CMD. Accordingly, considering the established relevance of sex-based mechanisms in MINOCA, sex was purposely included as an adjustment factor in our multivariate models to enhance the robustness of the observed association between fibrinogen and MINOCA.

### 4.6 Clinical Implications, Limitations, and Future Directions

We found that lower plasma fibrinogen levels were independently associated with the occurrence of MINOCA. This inverse relationship, which differs from the well-described positive association of fibrinogen with obstructive CAD and MI, suggests that fibrinogen may reflect a distinct biological phenotype within the MINOCA spectrum, in which inflammation and thrombotic load are qualitatively different. Further research into the causal role of fibrinogen across different pathophysiological subtypes of MINOCA could provide new perspectives, potentially supporting a fibrinogen-driven approach to more individualized clinical management within this entity of AMI.

Several limitations must be acknowledged. First, the study employed a retrospective, single-center design with a moderate sample size, which may limit the generalizability of our findings. Second, the lack of intracoronary imaging (e.g., OCT, IVUS) limited the investigation of mechanisms underlying individual cases of MINOCA to determine whether the association of fibrinogen differs across specific pathophysiological subtypes. Third, the lack of serial fibrinogen measurements limited the assessment of its dynamic changes and potential causal links with MINOCA.

Future studies should include larger, prospective, multicenter cohorts to validate our findings. In addition, evaluating longitudinal changes in fibrinogen and their associations with distinct pathophysiological subtypes of MINOCA may provide deeper mechanistic insights and help clarify causal relationships. Such knowledge could ultimately support more personalized and mechanism-based management strategies for MINOCA patients. Furthermore, the development of multi-marker models incorporating fibrinogen and other readily available clinical parameters may enhance the early recognition of MINOCA in routine practice.

## 5. Conclusions

In this retrospective study, lower plasma fibrinogen levels were independently associated with the occurrence of MINOCA, and this inverse relationship remained robust after multivariable adjustment and across clinical subgroups. Although fibrinogen alone shows limited discriminatory power, its biological plausibility suggests that it may serve as an adjunctive biomarker for the early recognition of MINOCA.

## Availability of Data and Materials

The datasets in our study are available from the corresponding author upon reasonable request.

## Supplementary Material

Supplementary material associated with this article can be found, in the online version, at https://doi.org/10.31083/RCM42845.

## References

[1] DeWood MA, Spores J, Notske R, Mouser LT, Burroughs R, Golden MS (1980). Prevalence of total coronary occlusion during the early hours of transmural myocardial infarction. *The New England Journal of Medicine*.

[2] Tamis-Holland JE, Jneid H, Reynolds HR, Agewall S, Brilakis ES, Brown TM (2019). Contemporary Diagnosis and Management of Patients With Myocardial Infarction in the Absence of Obstructive Coronary Artery Disease: A Scientific Statement From the American Heart Association. *Circulation*.

[3] Safdar B, Spatz ES, Dreyer RP, Beltrame JF, Lichtman JH, Spertus JA (2018). Presentation, Clinical Profile, and Prognosis of Young Patients With Myocardial Infarction With Nonobstructive Coronary Arteries (MINOCA): Results From the VIRGO Study. *Journal of the American Heart Association*.

[4] Smilowitz NR, Mahajan AM, Roe MT, Hellkamp AS, Chiswell K, Gulati M (2017). Mortality of Myocardial Infarction by Sex, Age, and Obstructive Coronary Artery Disease Status in the ACTION Registry-GWTG (Acute Coronary Treatment and Intervention Outcomes Network Registry-Get With the Guidelines). *Circulation. Cardiovascular Quality and Outcomes*.

[5] Agewall S, Beltrame JF, Reynolds HR, Niessner A, Rosano G, Caforio ALP (2017). ESC working group position paper on myocardial infarction with non-obstructive coronary arteries. *European Heart Journal*.

[6] Pasupathy S, Air T, Dreyer RP, Tavella R, Beltrame JF (2015). Systematic review of patients presenting with suspected myocardial infarction and nonobstructive coronary arteries. *Circulation*.

[7] Karakayali M, Altunova M, Yakisan T, Aslan S, Omar T, Artac I (2024). The Relationship between the Systemic Immune-Inflammation Index and Ischemia with Non-Obstructive Coronary Arteries in Patients Undergoing Coronary Angiography. *Arquivos Brasileiros De Cardiologia*.

[8] Karakayalı M, Altunova M, Yakışan T, Aslan S, Artaç İ, Omar T (2024). Relationship Between Nonobstructive Coronary Arteries and Metabolic Parameters. *Kafkas Journal of Medical Sciences*.

[9] Gao XY, Zhou BY, Zhang MZ, Zhao X, Qing P, Zhu CG (2017). Association between fibrinogen level and the severity of coronary stenosis in 418 male patients with myocardial infarction younger than 35 years old. *Oncotarget*.

[10] Zhou ZF, Hu CF, Gao MF, Jin X, Chen Y, Wang D (2024). The predictive value of serum fibrinogen and platelet distribution width for long-term cardiac death in acute myocardial infarction patients. *Journal of Thoracic Disease*.

[11] Cui Z, Zhao G, Liu X (2022). Blood fibrinogen level as a biomarker of adverse outcomes in patients with coronary artery disease: A systematic review and meta-analysis. *Medicine*.

[12] Thygesen K, Alpert JS, Jaffe AS, Chaitman BR, Bax JJ, Morrow DA (2018). Fourth Universal Definition of Myocardial Infarction (2018). *Journal of the American College of Cardiology*.

[13] Austen WG, Edwards JE, Frye RL, Gensini GG, Gott VL, Griffith LS (1975). A reporting system on patients evaluated for coronary artery disease. Report of the Ad Hoc Committee for Grading of Coronary Artery Disease, Council on Cardiovascular Surgery, American Heart Association. *Circulation*.

[14] Reynolds HR, Srichai MB, Iqbal SN, Slater JN, Mancini GBJ, Feit F (2011). Mechanisms of myocardial infarction in women without angiographically obstructive coronary artery disease. *Circulation*.

[15] Sara JD, Widmer RJ, Matsuzawa Y, Lennon RJ, Lerman LO, Lerman A (2015). Prevalence of Coronary Microvascular Dysfunction Among Patients With Chest Pain and Nonobstructive Coronary Artery Disease. *JACC. Cardiovascular Interventions*.

[16] Ong P, Athanasiadis A, Hill S, Vogelsberg H, Voehringer M, Sechtem U (2008). Coronary artery spasm as a frequent cause of acute coronary syndrome: The CASPAR (Coronary Artery Spasm in Patients With Acute Coronary Syndrome) Study. *Journal of the American College of Cardiology*.

[17] Prakash R, Starovoytov A, Heydari M, Mancini GBJ, Saw J (2016). Catheter-Induced Iatrogenic Coronary Artery Dissection in Patients With Spontaneous Coronary Artery Dissection. *JACC. Cardiovascular Interventions*.

[18] Pu J, Mintz GS, Biro S, Lee JB, Sum ST, Madden SP (2014). Insights into echo-attenuated plaques, echolucent plaques, and plaques with spotty calcification: novel findings from comparisons among intravascular ultrasound, near-infrared spectroscopy, and pathological histology in 2,294 human coronary artery segments. *Journal of the American College of Cardiology*.

[19] Zeng M, Zhao C, Bao X, Liu M, He L, Xu Y (2023). Clinical Characteristics and Prognosis of MINOCA Caused by Atherosclerotic and Nonatherosclerotic Mechanisms Assessed by OCT. *JACC. Cardiovascular Imaging*.

[20] Williams MGL, Liang K, De Garate E, Spagnoli L, Fiori E, Dastidar A (2022). Peak Troponin and CMR to Guide Management in Suspected ACS and Nonobstructive Coronary Arteries. *JACC. Cardiovascular Imaging*.

[21] Huang Y, Wang J, Guo Y, Shen L, Li Y (2024). Fibrinogen binding to activated platelets and its biomimetic thrombus-targeted thrombolytic strategies. *International Journal of Biological Macromolecules*.

[22] Ząbczyk M, Undas A (2017). Plasma fibrin clot structure and thromboembolism: clinical implications. *Polish Archives of Internal Medicine*.

[23] Stepien K, Nowak K, Wypasek E, Zalewski J, Undas A (2019). High prevalence of inherited thrombophilia and antiphospholipid syndrome in myocardial infarction with non-obstructive coronary arteries: Comparison with cryptogenic stroke. *International Journal of Cardiology*.

[24] Pasupathy S, Rodgers S, Tavella R, McRae S, Beltrame JF (2018). Risk of Thrombosis in Patients Presenting with Myocardial Infarction with Nonobstructive Coronary Arteries (MINOCA). *TH Open: Companion Journal to Thrombosis and Haemostasis*.

[25] Khorasani N, Mohammadi Y, Sarpoli M, Kazemi T, Riahi SM (2025). Understanding Myocardial Infarction with Non-Obstructive Coronary Arteries (MINOCA): a comprehensive meta-analysis of clinical characteristics, management, and prognosis compared to MI with the Obstructive Coronary Artery (MIOCA). *BMC Cardiovascular Disorders*.

