# Supplementary material for: Association Between Plasma Fibrinogen Level and the Risk of Myocardial Infarction With Non-Obstructive Coronary Arteries: A Retrospective Observational Study
Source: Rev Cardiovasc Med. 2026 Jan 19;27(1):42845. doi: 10.31083/RCM42845 (PMC12873661; doi:10.31083/RCM42845)
Supplement: Supplementary file 1 [file 2153-8174-27-1-42845-s1.zip › Supplementary Material.docx]

**Supplementary information:**

**Association between plasma fibrinogen level and the risk of MINOCA: a retrospective observational study**

Supplementary Table 1 Definitions and enrollment criteria for the study population

| Category | Specification |
| --- | --- |
| Definition of MINOCA | - Diagnosis meeting the universal criteria for AMI |
|  | - Non-obstructive coronary arteries on angiography (stenosis < 50% or normal) |
|  | - No clinically overt alternative cause of presentation |
|  |  |
| Definition of MI-CAD | - Diagnosis meeting the universal criteria for AMI |
|  | - Obstructive CAD on angiography (stenosis ≥50% in ≥1 major epicardial artery) |
|  | - No alternative non-ischemic explanation |
|  |  |
| Inclusion Criteria | - Age ≥18 years |
|  | - Diagnosis meeting the universal criteria for AMI |
|  | - Underwent coronary angiography during index hospitalization |
|  |  |
| Exclusion Criteria | - Duplicate hospitalizations or repeated data entries |
|  | - Known hematologic disorders |
|  | - History of thromboembolic disease or anticoagulant use within 3 months |
|  | - Severe hepatic or renal dysfunction  - History of organ transplantation |

AMI, acute myocardial infarction; MINOCA, myocardial infarction with non-obstructive coronary arteries; MI-CAD, myocardial infarction with obstructive coronary artery disease; CAD, coronary artery disease.


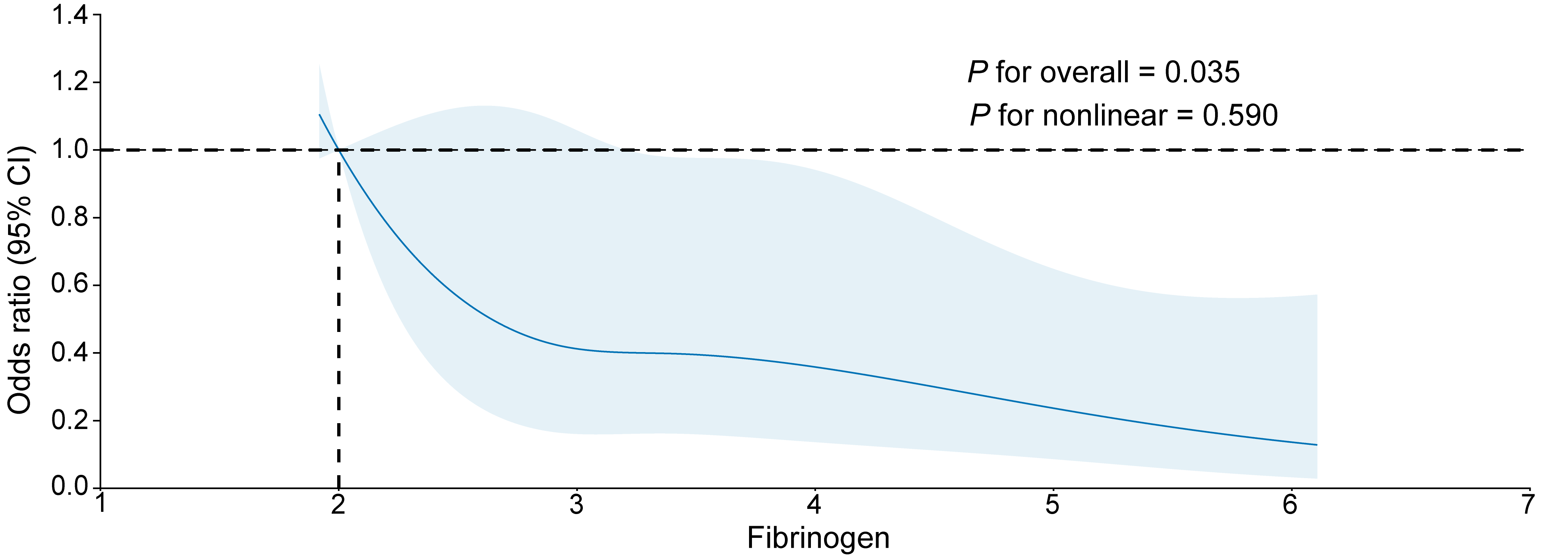


Supplementary Fig. 1. Restricted cubic spline analysis of the association between plasma fibrinogen level and the odds of MINOCA. The model was adjusted for age, sex, ST-segment elevation, diabetes mellitus, troponin I, and LVEF. MINOCA, myocardial infarction with non-obstructive coronary arteries; LVEF, left ventricular ejection fraction; CI, confidence interval.
